# Feeling Connected after Experiencing Digital Nature: A Survey Study

**DOI:** 10.3390/ijerph17186879

**Published:** 2020-09-21

**Authors:** Josca van Houwelingen-Snippe, Thomas J. L. van Rompay, Somaya Ben Allouch

**Affiliations:** 1 Communication Science, University of Twente, De Zul 10, 7522 NJ Enschede, The Netherlands; j.vanhouwelingen-snippe@utwente.nl; 2 Digital Life Research Group, University of Applied Sciences Amsterdam, Wibautstraat 2, 1091 GM Amsterdam, The Netherlands; s.ben.allouch@hva.nl

**Keywords:** digital nature, survey, loneliness, connectedness, social aspirations

## Abstract

Digital nature can provide a substitute for real nature for those who have limited access to green space, or are confined to their homes, for example during the worldwide COVID-19 lockdown. In a large-scale online survey, respondents (*N* = 1203) watched videos of digital nature, varying in terms of type of nature (wild versus tended nature) and spaciousness. Results show a significant increase of feelings of connectedness to the community after watching digital nature. Furthermore, tended nature scenes elicited more social aspirations than wild nature scenes. A multiple regression model further shows that living further away from nature was a significant predictor for loneliness scores, while number of nature interactions during a week was not. Results of this study confirm the importance of nature interaction for mental and social wellbeing for the general population and stress the potential of digital nature as a complementary strategy. These findings are of particular relevance to those who lack access to nature due to old age and related mobility constraints or a lockdown.

## 1. Introduction

Loneliness and social isolation are becoming increasingly recognized as serious public health concerns, with effects on both mental and physical health [1,2]. Especially during the (present) world-wide COVID-19 pandemic with the resulting lockdowns and social distancing regulations, loneliness and social isolation pose particularly urgent problems for a large part of the population [3,4,5]. Research suggests that feelings of connectedness are a better predictor of a number of health outcomes than the mere number of social contacts [6], suggesting that feelings of connectedness can play a key role in maintaining good (mental) health when social distancing is required.

A large body of research exists on the positive effects of nature interaction on mental wellbeing [7,8]; nature is fascinating and has the potential to restore attention [9], improve mood and positive emotions including awe [10,11,12] and even reduce feelings of loneliness and enhance feelings of social support [13]. Furthermore, people who feel related or connected to nature experience more feelings of happiness and feel generally more connected to the world at large [14]. On top of that, nature has been shown to promote a broad mind-set and increase creativity [15].

Nature, however, is not always around or accessible, for example for older adults with mobility restrictions or during (inter)national lockdowns. In situations where people have limited access to real life nature interaction [16], new technologies including virtual reality and augmented reality offer alternative opportunities to interact with nature. This is all the more interesting when considering research showing that interactions with simulated nature can have similar effects on mental health and wellbeing as interactions with real life nature [16,17,18].

However, so far research is silent on specific environmental features of simulated nature scenes involved [7]. Findings from a recent study [19] hint at the importance of spaciousness in the context of social wellbeing; spacious, rather than dense, digital nature scenes triggered more social aspirations. Additionally, different nature types may vary in the extent to which they are perceived as suitable for social contact. For instance, tended, rather than wild, scenes may more readily trigger associations with social interactions and feelings of togetherness. However, so far research has not investigated to what extent digital nature can increase feelings of connectedness and reduce feelings of loneliness.

In this study, an online survey was distributed via Amazon’s Mechanical Turk in which 1203 respondents watched a digital nature ‘walkthrough’ video, varying in terms of spaciousness and type of nature. The first research aim was to investigate whether experiencing digital nature (regardless of nature type and spaciousness) increases respondents’ feelings of connectedness to the community. Second, we aimed to investigate the effects of our experimental conditions, spaciousness and type of nature, on social aspirations, awe, fascination and sense of presence during the experience. We additionally aimed to investigate whether the mere presence of nearby nature reduces feelings of loneliness. Before elaborating on the details of the survey, the key notions involved will be discussed.

## 2. Theoretical Background

### 2.1. Nature and Social Wellbeing

An ever growing body of research underscores the potential of nature to improve social and mental wellbeing [7,8,9]. Nature is not only aesthetically pleasant, it has the potential to restore attention [9], increase perception of safety [8], increase positive mood and affect [7,15] and it can even accelerate recovery after surgery [20]. Generally, the amount of nearby public green space is associated with greater mental wellbeing [21] and walkable green space is associated with longevity of older adults in urban environments [22], further underscoring the importance of nearby nature.

Interestingly, a correlation has been found between perceived social support and the amount of green space in the living area [13]: Respondents who lived near (urban) green spaces experienced less feelings of loneliness, suggesting that the mere presence of nature can already have an effect on social wellbeing. Likewise, a positive relation has been found between loneliness and perceived distance to public parks and recreational areas [23]: People who experience nature as being closer at hand experience less feelings of loneliness compared to people who experience nature as more distant. Additionally, several studies show that nearby nature is positively correlated with feelings of connectedness and, in line with aforementioned study, reduces feelings of loneliness, while there was no such correlation with number of nature visits [24,25]. Additionally, nearby nature has a mediating role in the effect of social connectedness (i.e., the number of social contacts in the last week) on subjective wellbeing. In other words, respondents who scored low on social connectedness still reported high subjective wellbeing when they lived in an area with access to green space [25].

Research on psychological processes involved in nature interaction suggests that nature has the potential to connect people by promoting community-centred goals and the willingness to corporate with others [26], while urban environments stimulate self-centred goals and make people feel more selfish [27]. These combined findings stress the potential of nature interaction for enhancing social wellbeing and suggest that nearby nature is correlated with loneliness reduction and might enhance feelings of connectedness. However, there is only very limited research specifically addressing the relationship between nature and loneliness. In addition, and of particular relevance for present undertaking, research is silent on the extent to which digital nature may confer similar social benefits as real nature.

### 2.2. Digital Nature

As simulated nature might have similar effects on measures relevant to mental health as real nature [16,17,18], it can be applied in a variety of settings where there is a lack of (access to) real life nature interaction [16]. It has been shown that a short single-dose of nature videos in virtual reality can enhance positive mood levels [16]. Furthermore, immersive nature videos can evoke emotional responses and sense of presence that are comparable to responses evoked by real life nature experiences [28]. For older adults with mobility restrictions or for those with limited access to nature in general, virtual nature may therefore be used to promote health and to maintain a high quality of life [29], and it can even be used in therapy within psychiatric or medical care [30].

However, environments are multifaceted stimuli and experimental control over isolated variables is often impossible. As a result, insights in essential nature characteristics important for enhancing connectedness is limited. Adopting a virtual-reality approach allows for experimental investigation of nature characteristics. However, with the exception of one study [19], digital nature has not yet been used to create digital nature scenes systematically varying on specific nature characteristics. In the latter study (a lab experiment), four different conditions of simulated nature were tested, varying in terms of spaciousness and type of nature. Results showed that spacious nature projections elicited more social aspirations, and overall findings underscored the potential to enhance social wellbeing through simulated nature [19].

### 2.3. Variety in Types of Nature: Tended versus Wild Nature

In nature research, nature scenes are often contrasted with urban scenes, suggesting there is a global category of nature. However, nature comes in many forms and varies, amongst others, in terms of vegetation (the presence or absence of trees), absence or presence of water, and level of wildness (whether there are signs of civilization and human intervention such as walking paths and benches). Most studies on nature interaction report on the positive effects of wild nature, because of its opportunities for exploration and new experiences [9,31]. Additionally, wild and less predictable nature is often more mysterious [9,32,33], more fascinating [15] and provides a greater sense of “being away.” Research shows that wild forest scenes (displaying dense forest vegetation) had the largest restorative effect compared to other scenes varying in terms of wildness and density [34].

These findings suggest that higher restorative qualities are associated with more wild and natural scenes. However, there is only a small amount of studies that compare wild and tended nature, and often no differences between these conditions are found [19,35]. For example, previous research [36] revealed no significant differences in stress recovery across different nature types. However, a key word analysis indicated that wild nature scenes were described as more arousing than tended nature scenes.

In the context of social wellbeing and loneliness, tended nature scenes might be preferable as they come across as more inviting, more suitable for social interaction, and more readily trigger associations with safety and comfort. Man-made elements in an environment, such as benches, lanterns or planted flowerbeds, could give the impression of human intervention, in turn triggering associations with social contact in a safe setting (in line with [37]).

To sum up, understanding of what natural features are responsible for improvement of social wellbeing (including reductions in feelings of loneliness and increased feelings of connectedness) is very limited. As a result, it is still unclear whether wild or tended nature would be more suitable or appropriate for social interactions. Wild nature could act as a better conversation starter and might initiate social interaction more easily (in line with [38]). However, tended nature might be associated more readily with social presence and safety, and hence might be considered more suitable for social contact.

### 2.4. Spaciousness: Feeling Connected

Next to type of nature, spaciousness might well be a key variable. From an evolutionary perspective, spaciousness connects to the principle of being able to see (prospect) without being seen (refuge) as described in the prospect-refugee theory [39]. Findings from an experimental study showed that natural environments with high levels of prospect and low levels of refuge are restorative, while environments with low levels of prospect and high levels of refuge are not [40]. Additionally, for engagement of the mind, extent and scope of an environment are important [41]. More recent work also stresses the importance of spaciousness or vastness in landscapes [11,15].

Furthermore, research focusing on the emotion of ‘awe’, which occurs for example when watching vast or spacious nature scenes, is of particular interest since it stimulates pro-social behaviour [11]. Awe generally diminishes focus on the self and one’s personal concerns (small self), and establishes a more collective mind set (collective self) [11,42,43]. Participants who were exposed to an awe-eliciting experience were more willing to help others [44], and made more ethical choices [11]. Awe thus has the potential to distract attention away from everyday self-centred concerns towards the greater picture in life, thereby stimulating feelings of connectedness [10].

Awe can be elicited by a variety of experiences, however, nature interaction is a particularly important and prominent elicitor [43,45]. When participants were asked to recall a time when they experienced a ‘profound level of beauty’, most experiences involved vast nature and the experience of awe [45]. Nature experience thus has the potential to elicit awe and stimulate pro-social behaviours. Furthermore, since experienced vastness is an antecedent of awe, it could be expected that spacious landscapes elicit more awe than dense landscapes, and that they trigger a more collective mind-set (in line with [15]).

In a recent laboratory experiment, the experiential qualities of simulated spacious versus dense scenes with a static viewpoint were studied [19]. Spacious scenes elicited significantly more social aspirations, suggesting that spacious scenes are more suitable for social interaction with others. Findings further suggested that experiential contrasts across scenes (e.g., switching between extremes, such as moving from a dense to a spacious scene) can accentuate environmental qualities. In line with these findings, the current research presents walkthrough videos which take viewers along trough digital nature in which they travel from more open to dense scenes or vice versa.

### 2.5. Aim of Present Study

The first aim (Aim 1) of the present study is to investigate whether digital nature could provide an alternative to real nature (when real life nature interaction is not an option) for stimulating feelings of connectedness to one’s community. In other words, do people feel more connected after experiencing digital nature?

The second aim (Aim 2) is to investigate what characteristic features in nature contribute to feelings of social aspirations and related measures. Four digital nature conditions were developed, varying in terms of nature type (either wild or tended nature scenes) and spaciousness (transition from dense to spacious scenery, or vice versa), to investigate their effects on social aspirations, fascination, awe and sense of presence. We hypothesised that tended nature scenes elicit more social aspirations compared to wild nature scenes. Additionally, we hypothesised that the transition from a dense to a spacious nature scene elicits more social aspirations compared to a transition in the opposite direction.

The third aim (Aim 3) of the present study is to investigate the effects of living close to nature (measured in terms of the time it takes to walk to nearby nature) and the number of nature interactions during a week on feelings of loneliness.

## 3. Materials and Methods

The study has received approval from the ethics committee of the University of Twente, Faculty of Behavioural, Management and Social sciences (ethics code number: 200541).

### 3.1. Experimental Design

**Figure 1:**
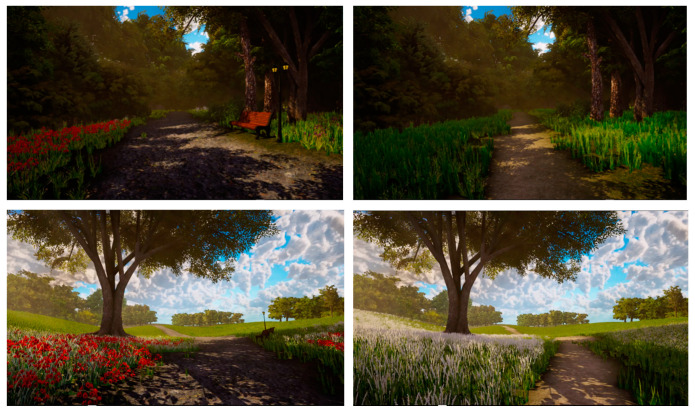
Screenshots of digital nature videos. From top left to bottom right: dense-tended nature, dense-wild nature, spacious tended nature, spacious wild nature.

A 2 × 2 between subjects design was used for this study, such that each respondent saw only one of the experimental condition videos, varying in terms of spaciousness (the video either displayed a transition from a dense to spacious scene, or a transition from a spacious to a dense scene) and type of nature (wild versus tended nature), see Figure 1.

### 3.2. Stimulus Development

Four different animated nature videos with auditory stimuli (soundtrack with bird sounds and sounds of footsteps) were developed using advanced game development software (Unity; Gaia Package; https://assetstore.unity.com/packages/tools/terrain/gaia-terrain-scene-generator-42618). All four videos consisted of a 30 s stationary viewpoint for the first scene, after which the viewpoint turned around (180 degrees turn) and followed a path through a virtual nature scene for 220 s to the final destination scene where the viewpoint remained stationary for another 30 s. The virtual environment further showed movement of trees, vegetation and corresponding shadows. The stimuli simulate interaction through movement and show a gradual transition from dense to spacious scenery or vice versa. (Source video files: Dense tended nature—spacious tended nature: https://vimeo.com/402730560. Spacious tended nature—dense tended nature: https://vimeo.com/402730696. Dense wild nature—spacious wild nature: https://vimeo.com/402730817. Spacious wild nature—dense wild nature: https://vimeo.com/402730945).

#### 3.2.1. Spaciousness Manipulation

Since contrasting scenes accentuated environmental qualities in a previous study [19], we decided to develop transitions (instead of switching from the first static scene to the second) between the spacious and dense scenes. In other words, the videos either started with a dense scene and (via a simulated walk along a path) gradually turned into a more spacious scene, or the other way around (following the same path). Screenshots of the spacious and dense scenes are presented in Figure 1.

#### 3.2.2. Type of Nature Manipulation

The videos either displayed a tended or a wild nature scene. The tended nature scenes displayed a park with lanterns, flowers and benches, while the wild nature scene displayed a less defined and narrower path with none of the objects indicating human intervention, see Figure 1.

### 3.3. Respondents

Respondents (*N* = 1203) were recruited online using Amazon’s Mechanical Turk in April 2020. Inclusion criteria concerned location: respondents in Northern European countries, Canada and the United States were included. Respondents who completed the survey were rewarded with 2$ (*N* = 1211), of which 8 were identified as outliers (based on standardised scores of > +−3 SD’s) and left out of the analyses.

### 3.4. Procedure

Before starting the survey, respondents filled out an informed consent form. Subsequently, a pre-intervention questionnaire was completed comprising demographic information, the nature-relatedness scale and the connectedness to community at large pre-test measure.

Next, the respondents watched the online digital nature intervention (i.e., the walkthrough video). They were instructed to turn on their sound and to watch the video full screen. After completing the video (participants could only proceed to the next page after watching the full-length video), manipulation checks were incorporated to verify that respondents actually managed to play the video as instructed. Subsequently, respondents were asked to fill-out the post-intervention questionnaire including the connectedness to community at large (post-test) measure and the other outcome measures. Following, respondents were asked whether they had comments on the digital nature videos. Finally, at the end of the survey, the respondents answered questions concerning their current living situation and the COVID-19 situation.

### 3.5. Measures

All measures were recorded using 5-point Likert scales, except for the single-item connectedness to community measure.

#### 3.5.1. Connectedness to Community

**Figure 2:**
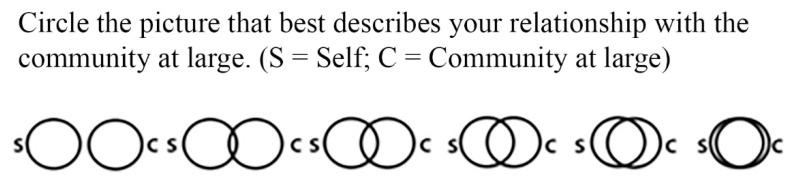
The inclusion of Community in the Self (ICS) Scale [46].

Connectedness to the community was measured using the Inclusion of Community in the Self Scale [46]. This single-item measure was used as a pre- and post-test; before and after experiencing the digital nature video, see Figure 2.

#### 3.5.2. Social Aspirations

Social aspirations were measured with the Social Aspiration Scale [19], including items such as ‘I would like to show this landscape to someone’ and ‘I would like to have a spontaneous chat here’. To increase construct validity, one item was deleted (α = 0.68 before deletion, α = 0.80 after deletion), resulting in four remaining items.

#### 3.5.3. Awe

Awe was measured using the constructs connectedness (i.e., ‘I felt closely connected to humanity’), self-diminishment (i.e., ‘I felt small compared to everything else’) and vastness (i.e., ‘I perceived something that was much larger than me’) of the Awe Experience Scale [47], consisting of 15 items (α = 0.96).

#### 3.5.4. Fascination

Fascination was measured using the fascination construct of the Perceived Restorativeness Scale [48], consisting of five items (α = 0.89), including items such as ‘I would like to spend more time looking at the surroundings’ and ‘My attention is drawn to many interesting things’.

#### 3.5.5. Loneliness

Loneliness was measured using the revised UCLA Loneliness scale [49], consisting of 20 items (α = 0.93) such as ‘I feel isolated from others’ and ‘There are people I feel close to (Reversed)’.

#### 3.5.6. Sense of Presence

Sense of Presence was measured using the self-location construct of the Spatial Presence Experience Scale [50], consisting of 8 items (α = 0.95) such as ‘I experienced the environment of the video as though I had stepped into a different place’ and ‘I felt as though I was physically present in the environment of the video’.

#### 3.5.7. Nature Relatedness

Nature relatedness was measured using the short version of the Nature Relatedness Scale [51], consisting of six items (α = 0.83) such as ‘My relationship to nature is an important part of who I am’ and ‘My ideal vacation spot would be a remote, wilderness area’.

## 4. Results

### 4.1. Demographic Information

Table 1 presents a summary of the demographic information of the respondents.

### 4.2. Connectedness Pre- Versus Post-Exposure (Aim 1)

Before and after experiencing the digital nature video, participants answered the single-item connectedness to the community at large measure. A Wilcoxon signed-rank test indicated that post-exposure connectedness scores were significantly higher (M = 3.94, SD = 1.27) than pre-exposure scores (M = 3.74, SD = 1.24), *Z* = −10.543, *p* < 0.001.

### 4.3. Effects of Experimental Conditions on Outcome Measures (Aim 2)

For each of the outcome measures (i.e., social aspirations, fascination, awe and sense of presence) a multiple regression was performed. Predictor variables were experimental conditions (spaciousness, type of nature and spaciousness*type of nature), demographics (year of birth, gender, country of residence, highest degree, employment status, marital status, living situation, living area, outdoor area, walking time, nature interactions and lockdown), trait measures (loneliness scores, nature relatedness scores and connectedness scores pre-exposure) and exposure characteristics (device, whether sound was turned on and whether the video was watched full-screen). The multiple regression models for social aspirations, fascination, awe and sense of presence are presented in Table 2. Only the effects of the experimental conditions on the outcome measures will be elaborated on in the following section.

#### 4.3.1. Social Aspirations

The variables predicted Social Aspirations, $F\left(22,1172\right)=14,51,\;p<0.001,\;R^{2}=0.21$, see Table 2. Type of nature was a significant (β = 0.086, *p* < 0.05) predictor for social aspiration scores, in such a way that the tended nature scenes scored higher than the wild nature scenes. Further confirming this outcome, a Mann Whitney U test indicated that the Social Aspiration scores were higher for tended nature scenes (M = 15.60, SD = 2.84) than for wild nature scenes (M = 15.09, SD = 3.21), U = 167610.5, *p* = 0.008.

Spaciousness and the interaction effect of Spaciousness * Type of nature were no significant predictors for Social Aspiration scores.

#### 4.3.2. Fascination

The variables predicted fascination scores, $F\left(21,1172\right)=21,54,\;p<0.001,\;R^{2}=0.28$, see Table 2. However, none of the experimental conditions added significantly to the prediction of fascination scores.

#### 4.3.3. Awe

The variables predicted awe scores, $F\left(21,1172\right)=39,27,\;p<0.001,\;R^{2}=0.41$, see Table 2. However, none of the experimental conditions added significantly to the prediction of awe scores.

#### 4.3.4. Sense of Presence

The variables predicted sense of presence scores, $F\left(22,1171\right)=25,20,\;p<0.001,\;R^{2}=0.32$, see Table 2. However, none of the experimental conditions added significantly to the prediction of sense of presence scores.

### 4.4. Effects of Demographics and Trait Measures on Loneliness (Aim 3)

A multiple regression was performed to predict the loneliness scores based on the demographic information and trait measures (nature relatedness and connectedness pre-exposure). The variables predicted loneliness scores, $F\left(14,1179\right)=17,33,\;p<0.001\;,\;R^{2}=0.17$ and are presented in Table 3.

Demographics

Marital status was a significant predictor for loneliness scores (β = 0.097, *p* < 0.05), showing that respondents who were married had lower loneliness scores compared to others.

Living situation was a significant predictor for loneliness scores (β = 0.099, *p* < 0.01), indicating that a high loneliness score is associated with living alone.

The time it takes respondents to walk to nature was also a significant predictor for loneliness (β = 0.301, *p* < 0.001), showing that respondents who lived further away from nature had higher loneliness scores.

Other demographic information (year of birth, gender, country of residence, highest degree, employment status, living area, outdoor area, nature interactions and lockdown) did not significantly add to the prediction of loneliness scores.

Trait measures

Connectedness pre-exposure was a significant predictor for loneliness scores (β = −0.271, *p* < 0.001): Respondents who felt connected to the community at large had a low loneliness score. Nature relatedness did not significantly add to the prediction of loneliness scores.

### 4.5. Qualitative Analysis of Comments on Videos

After filling out the post-exposure survey, respondents were asked whether they had comments on the digital nature videos. These qualitative comments were coded in Atlas.ti8. The majority of respondents who left comments were somehow positive about the experience in general (*n* = 332). The scenery was called beautiful (*n* = 95) and relaxing (*n* = 58). Some respondents even reported to be triggered to go into nature more often (‘I love nature and watching this video makes me want to go outside and explore and experience nature and its beauty’), or that this was the ideal input for people in lockdown situations (‘This is the type of video everyone who is quarantined needs to watch at least once a day in order to just stop worrying and enjoy the beauty of nature’). However, only a very small amount of the comments actually addressed the lockdown (*n* = 9), while the majority of respondents were experiencing some restrictions due to COVID-19, see Table 1. A number of respondents commented that the videos made them think about past nature experiences (‘I didn’t expect to be as impressed with [the] video as I was. My mind went back about 55 years ago when my family and I used to go to upstate New York in the summer which was much different than our apartment in the middle of New York City. Thank you for this nice experience.’) or even made them feel like they were actually walking down the path in the video (‘I felt like I was there walking down the path, looking and listening and enjoying the walk’). Some respondents even mentioned possible ways to use these videos in daily life, such as during meditation sessions or that it ‘would be nice to watch instead of taking a short walk at lunch if the weather was bad’.

More critical comments concerned the footstep sounds (*n* = 70) being either too loud, disruptive or mismatching the visuals. Respondents also commented on the level of realism, some reported that (parts of) the visuals were not realistic (*n* = 55), while others reported that (parts of) the visuals actually appeared realistic (*n* = 15). Additionally, there were comments on the quality of the visuals, however, these comments were both positive (*n* = 22) and negative (*n* = 23).

The bird sounds were consistent during all conditions, however, some respondents pointed out that when spaciousness varied, one would also expect to hear a difference in bird sounds. The bird sounds in general were mentioned as a positive feature of the videos (*n* = 45). Further possible improvements (*n* = 48) that were mentioned often concerned adding visual wildlife.

## 5. Discussion

In the present study, results of an online survey were reported in which respondents watched one of four digital nature video conditions, varying in terms of spaciousness and type of nature. The survey included measures for connectedness (pre- and post-experience), social aspirations and awe. The first aim of this study was to investigate whether connectedness scores increase after experiencing digital nature. Findings suggest that digital nature indeed has the potential to increase feelings of connectedness: after experiencing digital nature, respondents felt more connected to the community at large, which is in line with the research findings on interactions with real life nature [14]. In other words, the findings of the present study suggest that even a short dose of digital nature can relieve feelings of isolation by enhancing feelings of connectedness. Extending previous findings showing that a short dose of digital nature can positively impact attention and mood [16], our present findings indicate that digital nature can improve social wellbeing as well.

The second aim of this study was to investigate which specific nature characteristics can stimulate social aspirations and related measures. We hypothesised that tended nature scenes elicit more social aspirations compared to wild nature scenes. Findings indeed show a significant main effect of type of nature on social aspirations, suggesting that tended nature scenes elicit more social aspirations than wild nature scenes do. Tended nature scenes seem to provide affordances for pleasurable social interactions through objects such as benches and lampposts. However, type of nature was not a significant predictor for fascination, awe and sense of presence.

Additionally, we hypothesised that transitions from a dense to a spacious nature scene would elicit higher levels of social aspirations compared to transitions in the opposite direction. However, contrary to previous research [19], our findings do not provide support for this hypothesis. This might be due to the nature of the spaciousness manipulation. That is, rather than presenting participants with either a static spacious scene followed by an abrupt switch to a dense scene or vice versa, in the current study respondents experienced (while ‘walking’ through nature) a smooth and rather subtle transition from a dense to a more open scene or vice versa. This makes the difference between the spaciousness conditions less salient and makes it overall harder to pinpoint relationships between spaciousness, as manipulated in the current study, and outcome measures.

The third aim of the present study was to investigate the effects of living close to nature (i.e., the time it takes to walk to nearby nature) and the number of nature interactions during a week on feelings of loneliness. Findings show that living far away from nature was a predictor of loneliness scores, while number of nature interactions during a week was not, which is in line with previous findings [13,24,52]. As such, the results of the present study confirm findings from only a very limited amount of studies that assessed the relationship between nearby nature and loneliness explicitly. Other significant predictors for loneliness scores, such as living alone and being unmarried, are also in line with previous findings [53].

The present survey was distributed during the worldwide pandemic of COVID-19 and resulting (inter)national lockdowns. This was an ideal situation to reach a large number of respondents who were forced to stay inside. However, the results are not only restricted to those rare situations. When asked about the videos, only a small number of respondents actually mentioned the lockdown, whereas many respondents mentioned feeling relaxed after experiencing the digital nature videos or generally describing the experience as pleasant. Digital nature might also be used when weather conditions render a walk in nature less pleasant or for mediation and mindfulness purposes, as suggested by both previous research [35], and by comments from our participants. So, digital nature is not only of value for people with restricted access to nature or mobility restrictions but can complement outdoor experience in several ways.

### Limitations

The fact that no effect of spaciousness was found in the present study, while such an effect was found in previous work [19], might be due to the differences in presentation format; instead of immersive presentations, we used online videos that respondents watched on their own device at home. Additionally in the lab experiment, a large presentation setup with a broad video resolution (32 × 9) was used to enhance immersion [19]. We aimed to control for screen size by asking respondents to watch the videos full screen and asked them afterwards if they managed to do so. Nonetheless, we cannot rule out that participants did not deviate from our instructions. Additionally, screen size (for which we could not control) might influence immersion and arguably the perception of vastness in particular.

The qualitative analysis of comments yielded additional insights for improving the videos. Especially the sound of footsteps requires improvement in future iterations, as this was mentioned most often. Additionally, some technical issues (e.g., Wi-Fi or network issues) could have resulted in poor perceived video quality, however, overall the videos received positive feedback.

For future research, incorporating a control condition (a limitation of the present research) would allow for more definite conclusions when it comes to the role of digital nature in stimulating social connectedness.

Our findings are silent when it comes to differences in specific needs and requirements for digital nature across participants. For instance, needs and requirements for nature interventions may not only differ depending on age (e.g., adults versus children [54]), but may also vary depending on whether access to nature is temporarily limited (such as during the present COVID-19 crisis) or when access to nature is limited in general (e.g., for elderly with mobility restrictions). In light of previous findings [19], such differences might reflect variations in preferences for tended or wild nature scenes and corresponding needs for safety and comfort, which arguably are more salient for people with permanent mobility restrictions.

Finally, although our sample shows considerable variation in terms of demographics, it should be kept in mind that our sample is not representative of the general population as it consists of people interested in completing ‘intellectual’ tasks for a small reward. As such we cannot rule out differences in, for instance, intelligence and educational level.

## 6. Conclusions

The results of the present study suggest that even a short dose of digital nature can increase feelings of connectedness. Our findings further suggest that digital nature may not only be considered an alternative when outdoor nature is not available or accessible. That is, digital nature might also complement outdoor nature experience, especially when considering that digital nature allows for highlighting essential nature features. Future research should further pinpoint essential nature characteristics important for social connectedness and mental wellbeing.

## Figures and Tables

**Table 1:** Demographics profile.

Category	Specification	Number of Respondents
Year of birth	<1950	16
	1950–1959	73
	1960–1969	142
	1970–1979	231
	1980–1989	432
	1990–1999	299
	2000–2002	10
Gender	Male	775
	Female	425
	Other	3
Country of residence	USA	1093
	Canada	21
	North West Europe	89
Highest degree	High school or less	97
	Some college but no degree	170
	Associate degree	119
	Bachelor’s degree	527
	Master’s degree	258
	Professional or Doctoral degree	32
Employment status	Employed full time	817
	Employed part time	102
	Unemployed, unable to work, retired or homemaker	149
	Student	25
	Self-employed	106
Marital status	Married	623
	Single or never married	477
	Divorced or separated	86
	Widowed	17
Living situation	Alone	297
	With others	906
Living area	Rural	344
	Urban	859
Outdoor area	Garden	572
	Balcony	250
	Garden and balcony	147
	None	234
Walking time towards	0–9	428
nearby nature (in min)	10–19	313
	20–29	144
	30–39	140
	40–49	102
	50–60	76
Number of nature	Less than once	97
interactions during a week	1–2 times	589
	3–5 times	359
	More than 5 times	158
COVID-19 Lockdown	Total lockdown	366
	Partial lockdown	598
	Personal quarantine	193
	Other	46

**Table 2:** Multiple regression models for social aspiration, fascination, awe and sense of presence (* *p* < 0.05, ** *p* < 0.01, *** *p* < 0.001).

	Social Aspirations			Fascination			Awe			Sense of Presence		
Effect	B	SE	β	B	SE	β	B	SE	β	B	SE	β
(Constant)	8.953	5.209		12.222	5.985	*	18.418	19.238		16.245	12.544	
**Experimental conditions**												
Type of nature	0.519	0.226	0.086 *	0.203	0.259	0.028	−0.452	0.833	-0.017	0.028	0.543	0.002
Spaciousness	0.100	0.227	0.016	−0.272	0.260	−0.037	−0.758	0.837	−0.029	−0.491	0.546	−0.031
Type of nature * Spaciousness	0.123	0.319	0.018	0.360	0.366	0.043	1.376	1.178	0.046	0.938	0.768	0.052
**Demographics**												
year of birth	0.001	0.003	0.011	0.001	0.003	0.006	−0.003	0.009	−0.007	−0.002	0.006	−0.006
gender	0.038	0.171	0.006	0.099	0.196	0.013	0.971	0.631	0.036	0.739	0.411	0.045
country of residence	0.004	0.003	0.041	0.007	0.003	0.055 *	0.010	0.023	0.023	0.011	0.007	0.041
highest degree	0.105	0.068	0.044	0.078	0.078	−0.001	0.177	0.252	0.017	−0.051	0.164	−0.008
employment status	−0.052	0.035	−0.042	−0.071	0.078	−0.047	−0.164	0.128	−0.031	−0.132	0.083	−0.041
marital status	0.030	0.041	0.022	0.051	0.047	0.032	−0.243	0.150	−0.042	−0.147	0.098	−0.042
living situation	−0.530	0.201	−0.076 **	−0.497	0.231	−0.059 *	−0.951	0.744	−0.032	−0.322	0.485	−0.018
living area	0.014	0.091	0.004	0.091	0.105	0.022	0.072	0.337	0.005	−0.192	0.220	−0.022
outdoor area	−0.049	0.091	−0.015	−0.193	0.105	−0.048	0.003	0.336	0.0002	−0.193	0.219	−0.022
walking time towards nature	0.007	0.006	0.037	−0.003	0.007	−0.014	0.115	0.021	0.142 ***	0.049	0.014	0.100 **
nature interactions	−0.367	0.108	−0.099 **	−0.233	0.124	−0.052	−1.568	0.398	−0.099 ***	−0.651	0.260	−0.068 *
lockdown	−0.104	0.096	−0.029	−0.150	0.111	−0.035	−1.018	0.355	−0.016 **	−0.593	0.232	−0.063 *
**Trait measures**												
loneliness scores	−0.027	0.007	−0.112 ***	−0.036	0.008	−0.124 ***	−0.017	0.026	−0.016	−0.048	0.017	−0.075 **
nature relatedness scores	0.194	0.021	0.279 ***	0.328	0.024	0.391 ***	1.229	0.079	0.411 ***	0.596	0.051	0.329 ***
connectedness scores pre−exposure	0.448	0.079	0.183 ***	0.455	0.090	0.154 ***	2.773	0.291	0.264 ***	1.587	0.190	0.249 ***
**Exposure characteristics**												
device	−0.110	0.150	−0.020	−0.109	0.172	−0.016	−0.297	0.554	−0.012	−0.287	0.361	−0.020
sound turned on	−315	0.375	−0.023	−0.433	0.431	−0.026	−0.298	1.384	−0.005	0.065	0.903	0.002
full screen	0.017	0.166	0.003	−0.273	0.190	−0.037	−0.114	0.612	−0.004	−0.650	0.399	−0.041
**Effect**	**B**	**SE**	**β**	**B**	**SE**	**β**	**B**	**SE**	**β**	**B**	**SE**	**β**
	**Social Aspirations**			**Fascination**			**Awe**			**Sense of Presence**		

Type of nature: wild = 0, tended = 1. Spaciousness: spacious to dense = 0, dense to spacious = 1. Gender: male = 1, female = 2, other = 3. Country of residence: Austria = 10, Belgium = 17, Canada = 31, France = 61, Germany = 65, Netherlands = 122, UK = 185, USA = 187. Highest degree: less than high school = 1, high school graduate = 2, some college but no degree = 3, associate degree = 4, bachelor’s degree = 5, master’s degree = 6, doctoral degree = 7, professional degree = 8. Employment status: employed full time = 1, employed part time = 2, unemployed and currently looking for work = 3, unemployed and not currently looking for work = 4, student = 5, retired = 6, homemaker = 7, self-employed = 8, unable to work = 9. Marital status: married = 1, widowed = 2, divorced = 3, separated = 4, never married = 5, single = 6. Living situation: together with others = 1, alone = 2. Living area: urban = 1, rural = 2. Outdoor area: none = 0, garden = 1, balcony = 2, garden and balcony = 3. Number of nature interactions during a week: less than once a week = 1, 1–2 times a week = 2, 3–5 times a week = 3, more than 5 times a week = 4. Lockdown: total lockdown = 1, partial lockdown = 2, personal quarantine = 3, no regulations concerning COVID-19 = 4, Other = 5. Device: desktop computer = 1, laptop computer = 2, tablet = 3. Sound turned on: yes = 1, I don’t know = 2, no = 3. Full screen: yes = 1, I don’t know = 2, no = 3.

**Table 3:** Multiple regression model to predict Loneliness scores (* *p* < 0.05, ** *p* < 0.01, *** *p* < 0.001).

Effect	B	SE	β
(Constant)	51.422	21.715	*
**Demographics**			
year of birth	−0.005	0.011	−0.011
gender	−0.675	0.707	−0.026
country of residence	−0.004	0.011	−0.009
highest degree	0.423	0.285	0.043
employment status	0.006	0.144	0.001
marital status	0.537	0.168	0.097 *
living situation	2.855	0.836	0.099 **
living area	−0.142	0.383	−0.010
outdoor area	0.440	0.383	0.032
walking time towards nature	0.235	0.023	0.301 ***
nature interactions	0.020	0.452	0.001
lockdown	−0.120	0.404	−0.008
**Trait measures**			
nature relatedness scores	−0.032	0.089	−0.011
connectedness scores pre-exposure	−2.740	0.320	−0.271 ***

Gender: male = 1, female = 2, other = 3. Country of residence: Austria = 10, Belgium = 17, Canada = 31, France = 61, Germany = 65, Netherlands = 122, UK = 185, USA = 187. Highest degree: less than high school = 1, high school graduate = 2, some college but no degree = 3, associate degree = 4, bachelor’s degree = 5, master’s degree = 6, doctoral degree = 7, professional degree = 8. Employment status: employed full time = 1, employed part time = 2, unemployed and currently looking for work = 3, unemployed and not currently looking for work = 4, student = 5, retired = 6, homemaker = 7, self-employed = 8, unable to work = 9. Marital status: married = 1, widowed = 2, divorced = 3, separated = 4, never married = 5, single = 6. Living situation: together with others = 1, alone = 2. Living area: urban = 1, rural = 2. Outdoor area: none = 0, garden = 1, balcony = 2, garden and balcony = 3. Number of nature interactions during a week: less than once a week = 1, 1–2 times a week = 2, 3–5 times a week = 3, more than 5 times a week = 4. Lockdown: total lockdown = 1, partial lockdown = 2, personal quarantine = 3, no regulations concerning COVID-19 = 4, Other = 5. Device: desktop computer = 1, laptop computer = 2, tablet = 3. Sound turned on: yes = 1, I don’t know = 2, no = 3. Full screen: yes = 1, I don’t know = 2, no = 3.
